# Anti-Inflammatory Effects of *Solanum tuberosum* L. Polysaccharide and Its Limited Gene Expression Profile

**DOI:** 10.3390/ijms26125562

**Published:** 2025-06-10

**Authors:** Evgenii Generalov, Ilya Grigoryan, Vladislav Minaichev, Olga Sinitsyna, Leonid Yakovenko, Arkady Sinitsyn, Liubov Generalova

**Affiliations:** 1 Faculty of Physics, Lomonosov Moscow State University, 119991 Moscow, Russia; grigorian.iv19@physics.msu.ru (I.G.); yakovenko.lv@physics.msu.ru (L.Y.); 2 Institute of Theoretical and Experimental Biophysics, Russian Academy of Sciences, 142290 Pushchino, Russia; vminaychev@gmail.com; 3 Faculty of Chemistry, Lomonosov Moscow State University, 119991 Moscow, Russia; oasinitsyn@gmail.com (O.S.); apsinitsyn@gmail.com (A.S.); 4 Federal Research Centre ‘Fundamental of Biotechnology’ of the Russian Academy of Sciences (FRC Biotechnology RAS), 119071 Moscow, Russia; 5 Faculty of Medicine, Peoples’ Friendship University of Russia (RUDN University), 117198 Moscow, Russia; generals1100@mail.ru

**Keywords:** *Solanum tuberosum* L. polysaccharide, carrageenan-induced oedema, pocket granuloma, transcriptome, anti-inflammatory activity

## Abstract

Previous studies showed a potent anti-inflammatory activity of *Solanum tuberosum* L. polysaccharide (STP), which inhibited pro-inflammatory cytokines and stimulated anti-inflammatory ones in peptic ulcer models. Thus, the main goal of this study was to find out the molecular background of such activity and possible applications in different anti-inflammatory models. This study investigated the anti-inflammatory potential of the polysaccharide STP using model of LPS-induced inflammation in THP-1 macrophage-like cells (on the expression of *IL1B*, *IL6*, *IL10*, *TNF*, *NFKB1*, *BCL2*, *NRF2*, and *BAX*—genes involved in the regulation of inflammatory processes and oxidative stress), rat pocket granuloma, and carrageenan-induced oedema models. STP significantly reduced oedema volume, exhibiting a comparable anti-exudative effect to ibuprofen and surpassing the control group. The anti-inflammatory mechanism of STP extends beyond suppression of proinflammatory cytokine (*IL1B*, *IL6*, *TNF*) expression, as it also activates cellular defence mechanisms (*NRF2*, *BCL2*, *BAX*) and expression of anti-inflammatory cytokine (*IL10*). This complex, multifactorial action suggests that STP may possess significant therapeutic value for inflammatory conditions. The combined functional and molecular findings underscore STP’s potent anti-inflammatory properties, comparable to ibuprofen.

## 1. Introduction

Polysaccharides represent a broad class of natural carbohydrate polymers with significant biological activity, capable of modulating the immune response at multiple levels. Due to their unique structure, these compounds can influence various immune cells—such as macrophages, neutrophils, dendritic cells, and lymphocytes—by regulating their functions and balancing inflammatory responses [1,2]. They exert their effects through specific interactions with cell receptors, including toll-like receptors (TLRs), C-type lectin receptors (CLRs), and complement receptors [3]. These interactions activate signalling cascades, including the NF-κB and MAPK pathways. Depending on the chemical structure of the polysaccharide and the nature of receptor binding, the activated pathways may either enhance or suppress the inflammatory response.

A key effect of these compounds is their ability to modulate the cytokine profile. For example, β-glucans, which are found in mushrooms and cereals, can polarize macrophages toward an anti-inflammatory M2 phenotype [4]. This polarization is associated with reduced production of pro-inflammatory mediators—such as nitric oxide, prostaglandins, TNF-α, IL-1β, IL-6, and IL-12—while simultaneously increasing the levels of anti-inflammatory cytokines, like IL-10 and TGF-β, thus promoting the resolution of inflammation and aiding tissue regeneration [5].

The diversity of polysaccharide sources is reflected in their wide range of biological activities. Fungal polysaccharides, such as lentinan from shiitake mushrooms and β-glucans from reishi, exhibit potent immunomodulatory effects by activating macrophages and dendritic cells [6]. Plant-derived polysaccharides, including pectins from citrus fruits, arabinogalactans from larch, and rhamnogalacturonan from sugar beet, exert biological activity through distinct receptors [7,8]. Activation of TLR4, for instance, can lead to reduced expression of pro-inflammatory cytokines and stimulation of natural killer cells [9,10]. Polysaccharides from algae, such as fucoidan and laminaran, decrease leukocyte adhesion to the endothelium, regulate complement activity, and reduce tissue infiltration by inflammatory cells [11].

The structural features of polysaccharides, including branching degree, molecular weight, and the presence of specific functional groups, play a crucial role in determining their biological activity and receptor specificity. These features allow polysaccharides to exert either pro-inflammatory or anti-inflammatory effects depending on the context [12,13]. The broad impact of these compounds on the immune system makes them promising candidates for the development of novel immunomodulatory agents. Their ability to regulate the balance between pro-inflammatory and anti-inflammatory mechanisms makes them promising for the treatment of chronic inflammatory and infectious diseases, autoimmune disorders, and tissue regeneration following injury [14,15,16]. Polysaccharide modulation of pro- and anti-inflammatory pathways could be beneficial in treating brain inflammation [17,18] by influencing various intracellular signalling cascades, such as the NF-κB and PKC/PLC pathways [19,20], which play crucial roles in the pathogenesis of epilepsy [21,22]. Therefore, polysaccharides represent a promising class of compounds that not only correct pathological inflammatory responses but also promote tissue repair, making them significant for the development of modern therapeutic strategies.

Previous studies showed a potent anti-inflammatory activity of *Solanum tuberosum* L. polysaccharide (STP), which inhibited pro-inflammatory cytokines and stimulated anti-inflammatory ones in peptic ulcer models [15]. Thus, the main goal of this study was to find out the molecular background of such activity and possible applications in different anti-inflammatory models.

## 2. Results

### 2.1. In Vitro Study of Anti-Inflammatory Activity

**Figure 1:**
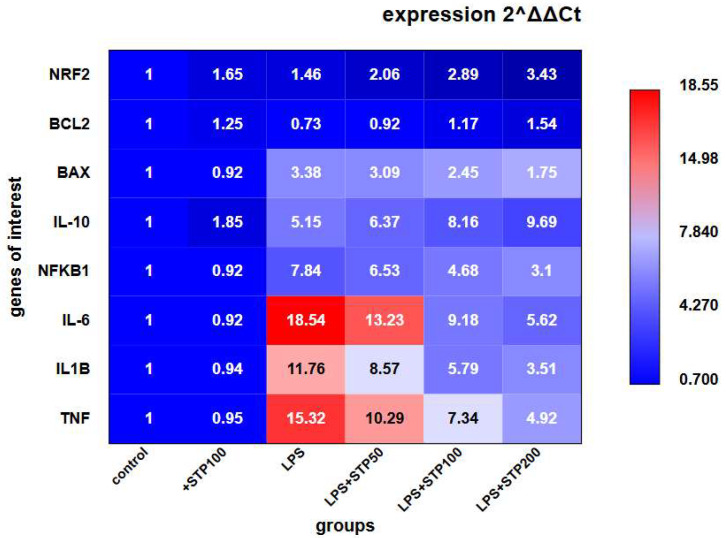
The effect of STP (50, 100, and 200 μg/mL) on the expression of genes involved in the regulation of inflammatory processes and oxidative stress in THP-1 macrophage-like cells under conditions of LPS-induced inflammation. mRNA levels were determined by real-time PCR. The vertical axis shows the studied genes, and the horizontal axis shows the experimental groups. The values in the cells represent fold changes in expression levels (2^ΔΔCt^) relative to control. *n* = 4.

We began the study by evaluating the anti-inflammatory activity of the polysaccharide STP in a model of LPS-induced inflammation in THP-1 macrophage-like cells. As shown in the heatmap (Figure 1), LPS stimulation resulted in a significant increase in the expression of pro-inflammatory genes: IL-6 increased by 18.54-fold, TNF by 15.32-fold, and IL1B by 11.76-fold compared to the control group, indicating a robust inflammatory response. Treatment with various concentrations of STP resulted in a dose-dependent reduction in the expression of these genes, with the highest concentration (200 μg/mL) reducing *IL6*, *TNF*, and *IL1B* levels to 5.62-, 4.92-, and 3.51-fold, respectively, relative to LPS stimulation.

Particular attention should be given to the dynamics of the anti-inflammatory gene *IL10*, which exhibited an inverse trend: as the concentration of STP increased, the level of *IL10* rose from 5.15-fold to 9.69-fold compared to LPS stimulation. This suggests that STP activates endogenous cellular mechanisms of anti-inflammatory protection.

In addition to its effects on the cytokine profile, STP treatment significantly influenced genes associated with oxidative stress and apoptosis. The data show that expression of *NRF2*—a key regulator of the antioxidant response—increased from 1.46-fold to 3.43-fold as the STP concentration increased. Changes in apoptosis-regulating genes were also observed: the anti-apoptotic gene *BCL2* demonstrated a dose-dependent increase (from 0.73-fold to 1.54-fold), while expression of the pro-apoptotic gene *BAX* decreased (from 3.38-fold to 1.75-fold). These findings suggest that STP exerts a protective effect, reducing cellular stress and enhancing cell viability under inflammatory conditions.

The data obtained demonstrate that the polysaccharide STP exerts a potent anti-inflammatory effect, not only by suppressing pro-inflammatory signals but also by activating cellular defence mechanisms through the upregulation of antioxidant and anti-apoptotic genes. The broad action of STP, confirmed by in vitro results in the LPS-induced inflammation model and further supported by in vivo experiments using carrageenan-induced oedema and pocket granuloma models in rats, highlights its potential as a promising therapeutic agent for the treatment of inflammatory diseases.

### 2.2. Carrageenan-Induced Oedema Model

**Figure 2:**
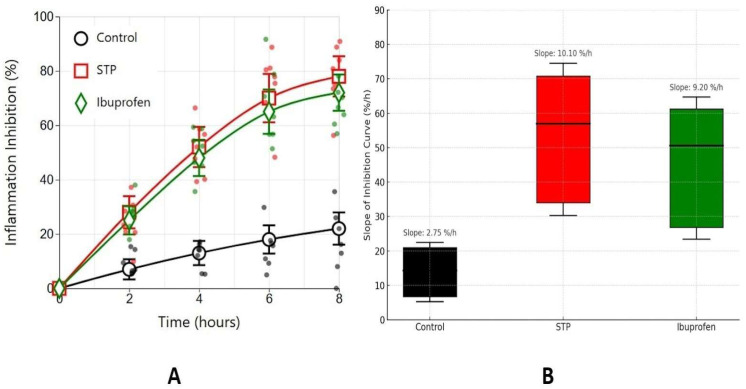
Time course of inflammation inhibition in rats’ carrageenan-induced oedema model. Red line—500 μg/rat STP administration; green line—100 mg/kg ibuprofen administration; black line—control with saline administration. (**A**) The time course of the inflammation inhibition index in animals. (**B**) Boxplot representation of the slopes of inflammation inhibition curves for each treatment group (Control, STP, and ibuprofen). The slope (%/h) reflects the rate of inflammation suppression over time, derived from linear regression of inhibition percentages across timepoints (0–8 h). Higher slopes indicate a faster onset of anti-inflammatory activity. The difference from the control is statistically significant *p* < 0.05 in STP and ibuprofen groups.

The anti-inflammatory activity of the polysaccharide STP was assessed in a carrageenan-induced oedema model in rats. Acute inflammation developed in the paw following the injection of carrageenan gel, as indicated by a significant increase in paw volume. To evaluate the effectiveness of STP, three experimental groups were established: a negative control group (animals received the vehicle with carrageenan), a positive control group (animals received ibuprofen, a standard non-steroidal anti-inflammatory drug), and an experimental group treated with the test solution of STP. Paw volume was measured dynamically over an 8-h period after inflammation induction, allowing for the assessment of both the degree of oedema inhibition (Figure 2A) and the rate of development of the anti-inflammatory effect (Figure 2B). The data presented in Figure 2 demonstrate that STP administration led to a significant reduction in paw volume increase. These results indicate that STP exhibits an anti-inflammatory effect, comparable to ibuprofen.

The STP group showed the steepest mean slope (10.10%/h), followed by the ibuprofen group (9.20%/h), while the control group exhibited minimal change (2.75%/h). Statistical analysis revealed significant differences between both STP vs. control and ibuprofen vs. control (*p* < 0.05, Bonferroni-corrected), as indicated by asterisks.

### 2.3. Air Pouch Granuloma Model

**Figure 3:**
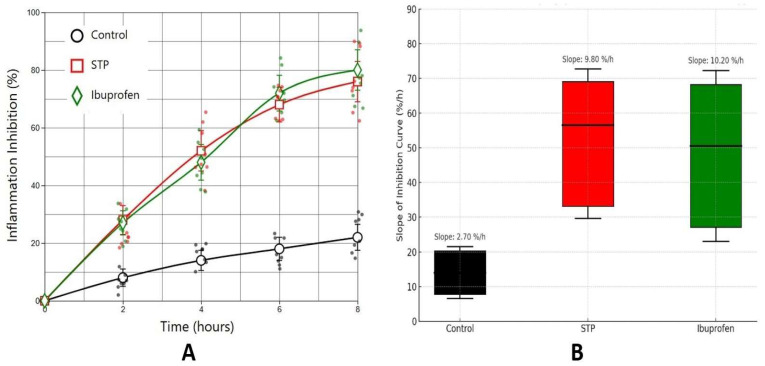
Time course of inflammation inhibition in rats’ pocket granuloma model. Red line—500 μg/rat STP administration; green line—100 mg/kg ibuprofen administration; black line—control with saline administration. (**A**) The time course of the inflammation inhibition index in animals. (**B**) Boxplot representation of the slopes of inflammation inhibition curves for each treatment group (control, STP, and ibuprofen). The slope (%/h) reflects the rate of inflammation suppression over time, derived from linear regression of inhibition percentages across timepoints (0–8 h). Higher slopes indicate a faster onset of anti-inflammatory activity. The difference from the control is statistically significant *p* < 0.01 in STP and ibuprofen groups.

Next, we evaluated the dynamics of inflammation suppression in the pocket granuloma model in rats by comparing the effects of STP with those of the positive control (ibuprofen) and the negative control (saline). Figure 3A shows that the time course of the inflammation inhibition index in animals treated with STP exhibited a significant increase in percentage inhibition compared to the control group, with no statistically significant differences between the STP and ibuprofen groups (*p* > 0.05). Thus, both treatments led to a substantial reduction in oedema volume, in contrast to the vehicle-treated group, where the inflammatory process remained pronounced.

Figure 3B illustrates the distribution of slope coefficients for each experimental group, reflecting the rate of development of the anti-inflammatory effect. As shown, the slope coefficients in the STP and ibuprofen groups are significantly higher than those in the control group (*p* < 0.001), indicating a faster and more effective suppression of inflammation following application of the active compounds.

The STP group showed a mean slope of 9.80%/h, comparable to ibuprofen (10.20%/h), while the control group exhibited minimal change (2.70%/h). Statistical analysis revealed significant differences between both STP vs. control and ibuprofen vs. control (*p* < 0.05, Bonferroni-corrected), as indicated by asterisks.

The slope of the inflammation inhibition curves was calculated for each treatment group using linear regression over the 0–8 h time course. The slope (%/h) reflects the rate at which anti-inflammatory activity increased over time. Both STP and ibuprofen demonstrated significantly steeper slopes compared to the control group, indicating a faster onset of action. Bars represent the interquartile range (IQR) with manually constrained whiskers (±10% IQR). Slope values are annotated above each group.

## 3. Discussion

The anti-inflammatory effect of the tested polysaccharide STP was evaluated in rats’ pocket granuloma and carrageenan-induced oedema models using both functional and molecular methods. The obtained data indicate that the administration of STP leads to a significant reduction in oedema volume, confirming the pronounced anti-exudative effect of the compound. When compared with the ibuprofen group, the dynamics of inflammation reduction in the STP group were comparable—no statistically significant differences were observed between them—whereas both active treatment groups significantly outperformed the control group that received only the vehicle with carrageenan.

To confirm the effect at the molecular level, the expression of key inflammatory genes was analysed by qPCR. The results showed a significant decrease in the levels of pro-inflammatory cytokines (IL-6, TNF-α, IL-1B) and a simultaneous increase in the expression of the anti-inflammatory gene *IL10* in animals treated with STP compared to the control group. These molecular data correlate with the functional outcomes, confirming that STP effectively modulates the inflammatory cascade by suppressing the production of pro-inflammatory mediators and activating protective mechanisms. Also, these results correlate with our previous works, where anti-inflammatory effects in peptic ulcers have been shown [15,23].

At the cellular level, STP decreased the expression of key pro-inflammatory mediators such as IL-6, TNF-α, and IL-1β. Suppression of these cytokines likely results from inhibition of the TLR4/NF-κB signalling cascade, a central pathway in inflammation induction [24,25]. Concurrently, STP elevated the expression of *IL10*, an essential anti-inflammatory cytokine known to regulate inflammation through the activation of the Jak/STAT3 signalling pathway, subsequently suppressing the production of pro-inflammatory mediators [26].

Another critical mechanism identified in our study was the activation of the antioxidant transcription factor NRF2. NRF2 regulates the expression of enzymes such as HO-1 and NQO1, which protect cells from oxidative stress-related damage [27,28]. Beyond antioxidant effects, NRF2 activation indirectly exhibits anti-inflammatory action by inhibiting NF-κB activity, thereby contributing to comprehensive tissue protection under inflammatory conditions.

Additionally, STP modulated the expression of apoptosis-regulating genes—*BCL2* (anti-apoptotic) and *BAX* (pro-apoptotic). The increased expression of BCL2 coupled with decreased *BAX* expression suggests a reduction in cellular apoptosis and preservation of tissue integrity under inflammatory stress, emphasising STP’s anti-inflammatory and cytoprotective effects.

Despite the promising results obtained, several significant limitations should be acknowledged:

Use of female animals only: It is known that biological sex substantially influences inflammation severity. Females typically exhibit less pronounced inflammation due to the anti-inflammatory properties of oestrogens [29,30]. Even under similar hormonal conditions, males generally demonstrate more intense inflammatory responses [31]. Furthermore, different routes of STP and ibuprofen administration can influence drug absorption, distribution, and metabolism. This could potentially affect the efficacy of the STP and ibuprofen comparison. Future studies should include the same routes of administration and both sexes to more accurately assess the universality and reproducibility of the observed effects.

Absence of protein-level validation: Gene expression analysis was conducted exclusively at the mRNA level. To fully confirm the functional relevance of the findings, further studies employing protein-based validation methods such as Western blotting or ELISA are required.

Short-term observation period (8 h): The brief duration of observation limits the evaluation of delayed effects and potential long-term side effects of STP, especially with chronic use.

In summary, the demonstrated effects highlight STP’s multi-level anti-inflammatory potential, involving cytokine regulation, antioxidant defence activation, and modulation of cellular survival mechanisms. This positions STP as a promising candidate for further investigations into therapies for chronic inflammatory diseases and conditions characterised by pronounced oxidative stress. Potential applications of STP include arthritis, inflammatory bowel disease, neuroinflammation, and other pathologies involving chronic inflammation and oxidative stress.

## 4. Materials and Methods

### 4.1. Studied Substance

This study examined the specific activity of the polysaccharide complex STP with a molecular weight around 70 kDa, consisting predominantly of arabinose and galactose, as well as other monosaccharides, including glucose. STP (obtained from LLC SPC Gemma-B, Moscow, Russia) was isolated and purified from *Solanum tuberosum* L. using cross-filtration, chromatography, and membrane purification methods according to the methodology described previously [16]. The selection of STP dosages for the studies (50, 100, and 200 µg/mL for in vitro and 500 µg/rat for in vivo) was based on preliminary experiments evaluating the toxicity and efficacy of the substance, as well as literature data on similar polysaccharide complexes.

### 4.2. In Vivo Study of Anti-Inflammatory Activity

#### 4.2.1. Experimental Animals

The study used female Wistar rats weighing 220–240 g (purchased from the branch of the Institute of Bioorganic Chemistry named after M.M. Shemyakin and Yu.A. Ovchinnikov RAS, Moscow, Russia), with 8 animals per group. The animals were housed in individually ventilated cages at a temperature of 22 ± 2 °C, relative humidity of 55 ± 10%, and a 12-h light/dark cycle, in accordance with the requirements of Directive 2010/63/EU of the European Parliament and of the Council of 22 September 2010, regulating the protection of animals used for scientific purposes [32].

All experimental protocols strictly adhered to the ethical principles outlined in the Declaration of Helsinki (1964) and its subsequent amendments. Animal studies were conducted in accordance with the guidelines of the Federation of European Laboratory Animal Science Associations (FELASA) [33] and the European Convention for the Protection of Vertebrate Animals (Strasbourg, 18 March 1986). The studies were approved by the Ethics Committee of the Institute of Cell Biophysics RAN (approval Nos. 4 dated 14 March 2022 and 3 dated 12 March 2023).

#### 4.2.2. Carrageenan-Induced Oedema Model

To assess the anti-inflammatory activity of STP, the carrageenan-induced oedema model [34], widely used for the primary screening of potential anti-inflammatory agents, was employed. The animals received a subplantar injection of 0.1 mL of 1% carrageenan gel into the right hind paw. The left hind paw, which did not receive the carrageenan injection, served as a control. STP solutions were administered subcutaneously into the interscapular region 1 h prior to the carrageenan injection.

Oedema intensity was measured at 4, 6, and 8 h after induction (time points chosen based on the dynamics of carrageenan-induced oedema, which reaches its maximum between 4–6 h and begins to subside at 8 h). The paw volume was measured using a digital plethysmometer (Ugo Basile, Gemonio, Italy) by immersing the paw up to the tarsal region in a water-filled measurement chamber. Each measurement was taken three times at 1-min intervals, and the average value was calculated. The inhibition index (*II*) was calculated using the formula:

(1) $$II=\frac{V_{k}-V_{o}}{V_{k}}$$

where *Vₖ* is the oedema volume of the control paw and *Vₒ* is the oedema volume of the treated paw.

The following groups were formed:

1. Control group—animals received a sterile 0.9% NaCl solution (*n* = 8);
2. Experimental group—animals received a sterile 0.9% NaCl solution containing STP at a dose of 500 µg/rat (*n* = 8);
3. Comparison group—animals received ibuprofen intraperitoneally at a dose of 100 mg/kg (*n* = 8).

#### 4.2.3. Air Pouch Granuloma Model

The air pouch granuloma model was also used to comprehensively evaluate the anti-inflammatory activity of STP at various stages of the inflammatory process [35]. The animals were administered 20 cm^3^ of sterile air subcutaneously in the interscapular region to form an air pouch, followed by the injection of 0.5 mL of a 50% oil solution of turpentine into the formed “pouch” to induce inflammation. Sterile STP solutions were administered subcutaneously into a different area 1 h prior to the turpentine injection.

Oedema was measured at 4, 6, and 8 h after inflammation induction. The volume of exudate in the granuloma was determined by puncturing and aspirating the fluid from the air pouch using a sterile syringe and needle, followed by volume measurement with a graduated tube. The inhibition index was calculated using the Formula (1) provided above.

The following groups were formed:

1. Control group—animals received a sterile 0.9% NaCl solution (*n* = 8);
2. Experimental group—animals received a sterile 0.9% NaCl solution containing STP at a dose of 500 µg/rat (*n* = 8);
3. Comparison group—animals received intraperitoneally ibuprofen at a dose of 100 mg/kg (*n* = 8).

### 4.3. In Vitro Study of Anti-Inflammatory Activity

#### 4.3.1. Cell Line and Culture Conditions

The study used the THP-1 cell line (human monocytes) obtained from the American Type Culture Collection (ATCC, catalogue number TIB-202, Manassas, VA, USA). The cells were cultured in RPMI-1640 medium supplemented with 10% foetal bovine serum (FBS), 2 mM L-glutamine, 100 U/mL penicillin, and 100 µg/mL streptomycin (all reagents from Gibco, Waltham, MA, USA). Cultures were maintained in a humidified atmosphere with 5% CO_2_ at 37 °C in a CO_2_ incubator (Thermo Fisher Scientific, Waltham, MA, USA).

#### 4.3.2. Differentiation of Monocytes into Macrophage-like Cells

To differentiate THP-1 monocytes into macrophage-like cells, suspension cultures (1 × 10^6^ cells/mL) were seeded into 6-well plates and treated with phorbol 12-myristate 13-acetate (PMA, Sigma-Aldrich, Saint Louis, MO, USA) at a concentration of 100 ng/mL for 96 h. After this period, the medium containing PMA was removed, and the cells were washed twice with phosphate-buffered saline (PBS) and left in fresh medium without PMA for 24 h to allow for recovery and stabilization of the macrophage-like phenotype.

#### 4.3.3. Induction of Inflammation and Treatment with the Investigated Substance

After the recovery period, the cells were treated with lipopolysaccharide (LPS from *E. coli* O111:B4, Sigma-Aldrich, USA) at a concentration of 100 ng/mL to induce an inflammatory response. Simultaneously, STP was added to the culture medium at concentrations of 50, 100, and 200 µg/mL (the concentrations were chosen based on preliminary cytotoxicity studies demonstrating the absence of toxic effects at these doses). Control cultures were treated with PBS (negative control) or with LPS alone (positive inflammation control). The cells were incubated in the presence of LPS and STP for 24 h in three biological replicates for each condition, after which samples were collected for subsequent gene expression analysis.

#### 4.3.4. Gene Expression Analysis by Real-Time Quantitative PCR

##### Primers Design

Specific primers for real-time quantitative PCR were designed using the online tool NCBI Primer-BLAST (https://www.ncbi.nlm.nih.gov/tools/primer-blast/ (accessed on 15 January 2025)). The design parameters included: oligonucleotide length of 18–25 bases, melting temperature of 58–62 °C, GC content of 40–60%, and an amplicon length of 80–150 base pairs. Primer specificity was confirmed using the BLAST algorithm to exclude the possibility of nonspecific amplification.

For the study, genes related to inflammatory processes (*TNF*, *IL1B*, *IL-6*, *NFKB1*, *IL10*), antioxidant protection (*NRF2*), and apoptosis (*BAX*, *BCL2*) were selected to evaluate the impact of STP on various aspects of the cellular response during inflammation. The sequences of the primers used are provided in Table 1.

#### RNA Extraction and Reverse Transcription

Total RNA was extracted from the samples using the phenol–chloroform extraction method, with a commercial reagent kit (Evrogen, Moscow, Russia) according to the manufacturer’s instructions. The quality of the extracted RNA was evaluated spectrophotometrically at wavelengths of 260 and 280 nm using a NanoDrop (Thermo Fisher Scientific, USA); an A260/A280 ratio > 1.8 was considered indicative of high purity. Additionally, RNA integrity was confirmed by electrophoresis in a 1% agarose gel.

Complementary DNA (cDNA) synthesis was performed using the MMLV reverse transcriptase (Evrogen, Russia). The reaction mixture (total volume 20 µL) contained 1–2 µg total RNA, oligo (dT) primers (20 µM), a mixture of deoxynucleotide triphosphates (10 mM), dithiothreitol (20 mM), and 100 units of reverse transcriptase. The reverse transcription reaction was carried out at 40 °C for 45 min, followed by enzyme inactivation at 70 °C for 10 min.

#### Real-Time Quantitative PCR

The obtained cDNA samples were used as templates for real-time quantitative PCR. The reaction mixture included 3 µL of cDNA (diluted 1:10), specific primers (10 µM each), and a commercial mix containing SYBR Green and a thermostable DNA polymerase (Evrogen, Russia). Amplification was performed on a DTlite device (DNA-Technology, Moscow, Russia) using the following program: initial denaturation at 95 °C for 5 min, then 40 cycles consisting of denaturation (95 °C for 30 s), primer annealing (temperature depending on the primers, 58–62 °C for 30 s), and elongation (72 °C for 30 s). Each sample was analysed in three technical replicates.

After amplification, melting curve analysis was performed (temperature range 65–95 °C with a 0.5 °C increment) to confirm the specificity of the PCR products.

#### Data Analysis and Quality Control

To exclude genomic DNA contamination, each experiment included a negative control without the addition of reverse transcriptase (RT) and a PCR negative control without template (NTC). The absence of a signal in these samples confirmed the purity of the RNA preparations and reaction mixtures.

To quantify the rate of development of the anti-inflammatory effect, we calculated the slope coefficient based on a linear approximation of the change in the inhibition index (*II*) over time. For each group of animals (*n* = 8), the *II* values at time points of 4, 6, and 8 h were approximated by a straight line using the least squares method. The slope coefficient of the resulting line reflected the rate of change (increase) in the anti-inflammatory effect of the drug. Calculations were performed using the built-in linear regression functions in the OriginLab 2024b package. The obtained slope coefficient values were then compared between the groups using the nonparametric Kruskal–Wallis test with the Bonferroni correction.

The relative expression levels of the target genes were determined using the 2^−ΔΔCt^ method. GAPDH, encoding glyceraldehyde-3-phosphate dehydrogenase, was used as the reference gene for normalization; its expression stability was confirmed under the experimental conditions [36,37]. The efficiency of PCR for each gene was calculated using serial dilutions of cDNA and ranged from 95–105%.

### 4.4. Statistical Analysis

Statistical analysis was performed using OriginLab 2024b software. The normality of the data distribution was tested using the Shapiro–Wilk test. Since most datasets did not follow a normal distribution (*p* < 0.05), non-parametric methods were used for further analysis. Group comparisons were performed using the Kruskal–Wallis test with subsequent Bonferroni correction for multiple comparisons. Results are presented as the median and interquartile range. Differences were considered statistically significant at *p* < 0.05. All experiments were repeated at least three times.

## 5. Conclusions

The presented data demonstrate the complex actions of STP, which is manifested not only in the suppression of proinflammatory cytokines, but also in the activation of cellular defence mechanisms through increased expression of anti-inflammatory factors and modulation of genes associated with oxidative stress and apoptosis. Such a multifactorial effect may be of significant importance for the potential therapeutic use of STP in inflammatory diseases.

In summary, the combined functional and molecular data indicate that the polysaccharide STP exhibits a pronounced anti-inflammatory effect in the pocket granuloma model, providing an effect comparable to ibuprofen and significantly exceeding the response observed in the control group. These results open up prospects for further investigation of the molecular mechanisms of STP action and its potential clinical application in the treatment of inflammatory diseases.

## Figures and Tables

**Table 1:** Primer sequences for real-time quantitative PCR.

Gene (Protein)	Forward Primer (5′→3′)	Reverse Primer (5′→3′)
*GAPDH*	GAAGGTGAAGGTCGGAGTC	GAAGATGGTGATGGGATTTC
*TNF* (TNF-α)	CCTCTCTCTAATCAGCCCTCTG	GAGGACCTGGGAGTAGATGAG
*IL1B* (IL-1β)	ATGATGGCTTATTACAGTGGCA	GTCGGAGATTCGTAGCTGGA
*IL6*	ACTCACCTCTTCAGAACGAAT	CCATCTTTGGAAGGTTCAGGTTG
*NFKB1*	AACAGAGAGGATTTCGTTTCC	TTTGACCTGAGGGTAAGACTTCT
*IL10*	GACTTTAAGGGTTACCTGGGT	TCACATGCGCCTTGATGTCTG
*NRF2*	TTCCCGGTCACATCGAGAG	TCCTGTTGCATACCGTCTAAATC
*BAX*	CCCGAGAGGTCTTTTTCCGAG	CCAGCCCATGATGGTTCTGAT
*BCL2*	GGTGGGGTCATGTGTGTGG	CGGTTCAGGTACTCAGTCATCC

## Data Availability

The data presented in this study are available on request from the corresponding author.

## Abbreviations

The following abbreviations are used in this manuscript:

STP	*Solanum tuberosum* L. polysaccharide
cDNA	Complementary DNA
IQR	Interquartile range
